# Allocating municipal services to individuals with complex rehabilitation needs – a discourse analysis of individual administrative decision letters

**DOI:** 10.1186/s12913-024-10972-7

**Published:** 2024-04-12

**Authors:** Maren Ekenes, Olin Oldeide, Eike Wehling

**Affiliations:** 1 Department of Physical Medicine and Rehabilitation, Haukeland University Hospital, Bergen, Norway; 2 Department of Health Promotion and Development, University of Bergen, Bergen, Norway; 3 Department of Biological and Medical Psychology, University of Bergen, Bergen, Norway

**Keywords:** Rehabilitation, User involvement, Coordination, Service allocation, Municipality, Acquired brain injury

## Abstract

**Background:**

Rehabilitation is considered paramount for enhancing quality of life and reducing healthcare costs. As a result of healthcare reforms, Norwegian municipalities have been given greater responsibility for allocating rehabilitation services following discharge from hospital. Individual decision letters serve as the basis for implementing services and they have been described as information labels on the services provided by the municipality. They play an important role in planning and implementing the services in collaboration with the individual applicants. Research indicates that the implementation of policies may lead to unintended consequences, as individuals receiving municipal services perceive them as fragmented. This perception is characterised by limited user involvement and a high focus on body functions. The aim of this study was to examine how municipal decision letters about service allocation incorporate the recommendations made in the official national guideline and reflect a holistic approach to rehabilitation, coordination and user involvement for individuals with comprehensive needs.

**Methods:**

The decision letters of ten individuals with moderate to severe brain injury allocating rehabilitation services in two municipalities were examined. It was assessed whether the content was in accordance with the authorities’ recommendations, and a discourse analysis was conducted using four tools adapted from an established integrated approach.

**Results:**

The letters primarily contained standard texts concerning legal and administrative regulations. They were predominantly in line with the official guideline to municipal service allocation. From a rehabilitation perspective, the focus was mainly on medically oriented care, scarcely referring to psychosocial needs, activity, and participation. The intended user involvement seemed to vary between active and passive status, while the coordination of services was given limited attention.

**Conclusions:**

The written decision letters did fulfil legal and administrative recommendations for service allocation. However, they did not fulfil their potential to serve as a means of conveying rehabilitation issues, such as specification of the allocated services, a holistic approach to health, coordination, or the involvement of users in decision processes. These elements must be incorporated throughout the allocation process if the policies are to be implemented as intended. Findings can have international relevance for discussions between clinicians and policy makers.

## Background

Worldwide, healthcare services are of great interest to policy-makers, service-providing organisations, and the individuals concerned [1, 2]. Rehabilitation has become an area of fundamental interest because of increased survival after even severe injuries and because it enhances the quality of life of individuals affected, as well as reducing healthcare costs [3, 4]. Discourses on rehabilitation, including how health and disability are understood, individually and socially, have changed during recent decades [5–9]. As a result of these changes, the conceptualisation of rehabilitation has shifted from primarily medically oriented care to holistic, socially oriented care that includes biological, psychological and social factors [10, 11]. The latter approach includes the individuals’ overall situation, e.g. body structures and functions, activities, participation, and contextual factors [5, 12, 13]. Despite these changes, healthcare systems often continue to predominantly deliver medically oriented care (which focuses on disease and symptoms) and pay little attention to socially oriented care (which focuses on participation, quality of life and social support) [14].

Patients with moderate to severe brain injury can be characterised as a group with comprehensive rehabilitation needs. Individuals may experience long-term physical, cognitive, behavioural and emotional challenges [15]. Rehabilitation may last for years, and is provided in many settings, i.e. in specialist health care, municipal institutions or at home [15, 16]. For individuals with comprehensive needs, complex rehabilitation pathways usually start in specialist health care before continuing at the municipal level [15, 17–20]. Previous research shows that individuals achieve better outcomes when rehabilitation is coordinated, and when it is provided in collaboration with patients and next of kin [15]. However, services have been described as lacking coordination, leaving patients and next of kin struggling through their rehabilitation pathways [11, 15, 16, 21].

Individual rights to participation and self-determination in rehabilitation processes have become paramount in healthcare discourses [5]. Thus, the services are to be aligned with the needs and values of the individual [22]. The individual’s role as user of services, while previously commonly described as passive, trusting, and adapting to professionals’ instructions, is now described as an active role with patients being responsible, knowledgeable, competent, information-seeking and able to take care of their own health [5, 23, 24]. However, various researchers have stressed that the changes towards an active user, despite originally good intentions, may have drawbacks [5, 23]. In rehabilitation settings, individuals constitute a heterogeneous group, demonstrating great diversity of physical, cognitive and social difficulties. Some may be unable to play an active role or cooperate, which, in turn, may lead to them receiving fewer or less suitable services [5].

In a national healthcare reform in Norway, municipalities were given greater responsibility for rehabilitation services as regards decision processes, and the scope and type of services provided for individuals in need of rehabilitation [25]. The reform was designed to enhance care coordination and use the resources within the Norwegian healthcare system more efficiently, i.e. by discharging patients earlier from hospitals to municipal care and rehabilitation [26]. The reform advocated a lower allocation to specialist health care and more focus on public-oriented practices in the municipalities [5]. Røberg et al. [5] identified that such a shift towards fewer specialist healthcare services could have unintended consequences in that it could lead to a more undetermined conceptualization of rehabilitation and a downscaling of individual rehabilitation services for individuals with comprehensive rehabilitation needs.

Municipal decisions on the allocation of in-patient services exceeding two weeks require a written individual administrative decision. The decision letter is described as an information label [27]. An official national guideline has been implemented to ensure high-quality case management, stressing fair and equal treatment, emphasising legal regulation of service provision, ensuring that service providers are accountable, that decisions are transparent and safeguard the user’s rights [28]. The importance of the written individual decisions is underscored as they directly affect what services are provided by healthcare staff to the individual [29, 30].

Only a few studies have analysed the content of decision letters concerning service allocation. In a national audit by the Norwegian Directorate of Health, decision letters were found to be too generally worded, and to lack individual or next of kin-related considerations [27]. Some authors found that letters adhered to the national guideline for service provision [31]. However, they suggested that the guideline should be more closely examined as regards fair and equal treatment in the allocation of services. Furthermore, an insufficient assessment of psychosocial needs among patients with dementia has been reported [32].

How the local level implements policy provides insights into how policy is translated into action by the organisations and service providers responsible for the execution of policy [33–35]. Hence, studying written decision letters may provide important information about how and to what extent municipal actors, in their implementation of policy, contribute to healthcare discourses. Internationally, there is a growing interest on how health care services are allocated and organised. Although, countries have different laws and guidelines, research on the implementation of national policies is international relevant as it may stimulate health care staff, researchers, and policy makers to reflect on how their systems work and what could be changed. In several countries it is good practice or a legal obligation to notify users in writing of administrative decisions [36]. Thus, analyses of individual administrative decision letters may contribute to national and international discourses of the content of written decisions and rehabilitation. In the present study, we aimed to examine how Norwegian municipal administrative decision letters concerning service allocation incorporate recommendations in the official national guideline of service allocation and whether they reflect a holistic approach to rehabilitation, coordination, and user involvement for individuals with comprehensive needs. By analysing the letters as policy implementation tools using discourse analysis, the article sheds light on how policies are translated into action.

## Methods

### Design

This article is a part of a larger case study investigating service allocation to individuals who need comprehensive municipal interdisciplinary rehabilitation in the transition from hospital rehabilitation to municipal rehabilitation in Norway [37]. Data comprising decision letters, electronic correspondence and interviews were collected over a four-year period during the past decade. The decision letters were analysed in this study.

### Participant characteristics and data collection

Letters to ten participants aged 18 to 80 years, who were admitted to hospital due to moderate or severe brain injury and spent between 7 and 28 weeks in a rehabilitation unit in specialist health care, were analysed. During their stay, they all received rehabilitation services from an interdisciplinary team comprising specialists in physical medicine and rehabilitation, nurses, occupational therapists, physiotherapists, social workers, speech and language therapists and neuropsychologists. At the point of discharge, all participants were regarded as needing further interdisciplinary municipal rehabilitation, individual plans and coordinated services.

Data collection was carried out in accordance with ethical guidelines for research, and the data processing procedures were approved by the Norwegian Centre for Research Data and the Hospital Data Protection Officer. The administrative municipal leadership granted approval for the study to be conducted. Participants with a moderate or severe brain injury may be unable to give informed consent due to an inability to communicate or markedly impaired cognitive abilities. This raises legal and ethical issues as regards informed consent [38–40], since it is an obligation and a cornerstone of medical ethics and health law that medical professionals obtain informed consent prior to medical treatment or research [41]. This principle acknowledges individuals’ autonomy, while at the same time protecting the individuals whose autonomy is reduced. Patients are legally competent to give informed consent when they have decision-making capacity as autonomous decision-makers. Decision-making capacity includes the ability to understand relevant information, to appreciate the consequences of treatment/participation in a research project, to discuss options, and to communicate a choice. When participants do not meet these criteria, medical professionals must seek informed consent from a substitute decision-maker who bases his/her judgement on the previously expressed wishes and preferences of the participant, or, if no wishes have been expressed, on the best interests of the participant [42]. In the current study, it was expected that not all participants would be able to consent. It was therefore discussed whether participants who lacked the capacity to give consent should be excluded. However, the exclusion of vulnerable populations from healthcare studies is seen as problematic, since knowledge about this diverse population would remain hidden [40, 43]. Hence, it was decided to include participants who needed comprehensive rehabilitation but who had reduced cognitive capacity and were not capable of granting informed consent. The risk and burden to the individual participant were regarded as low, while gaining knowledge about service allocation to this specific population was regarded as important. In the current study, three participants were evaluated as having the capacity to grant informed consent by the physician in charge. Of the remaining seven, informed consent was obtained from their next of kin/legal guardian. Pseudonyms were used and dates, municipalities, diagnoses and multimorbidity have been anonymised to ensure anonymity.

### Setting

The participants were discharged to two municipalities, two of them to a town municipality (inhabitants < 15 000; town municipality), the remaining eight to a city municipality (inhabitants > 100 000; city municipality. In both municipalities, decision letters were sent by the offices with power of decision, organised separately to the service providing units.

#### Official recommendation and municipal decision letters

The guideline states that the written decisions must include i) the services to be provided, the extent of services and the timeframe, ii) the justification for the decision, with complete and correct legal references, the facts the decision is based on and the main considerations regarding the exercise of discretion, iii) the right of access to services and the right to request deferred implementation of decision and reference to the right of appeal. Furthermore, it stresses the municipality’s responsibility for initiating individual plans and for coordinating services for individuals in need of comprehensive services. However, it is not mandatory to name all services in a written decision. With regard to the coordination of services, the guideline underlines that decisions concerning the different services may be included in the same letter to give an overview of the overall services. Further, it emphasises the legal regulations, which give great importance to the involvement of individual applicants and their next of kin in the allocation of services. It is emphasised that the decision must be detailed enough for the applicants and their next of kin to know what specifically is granted, and to evaluate whether the services are sufficient.

### Data analysis

Firstly, matrices were created to provide an overview and to compare and contrast each letter’s content based on the content categories recommended in the national guideline. Secondly, a discourse analysis based on Gee [44] was used to examine the letters, since this makes it possible to analyse how organisations and individuals construct and reconstruct meanings in written documents [44, 45]. This method was chosen because language builds identities, relationships and significances [44–46], which also apply to official documents [44–47]. Gee [48] suggest an overarching toolkit of 28 tools to be considered when conducting a discourse analyses. According to him, these tools provide questions for the researcher to “immerse” into the letters and can be adapted to the needs and demands of the individual study. In this study, we initially conducted an explorative analysis of the letters applying the 28 tools. We found four tools particular relevant. These four tools were adapted for further in-depth analysis in line with the relevance for the study [46, 48]. The four included tools were *the subject tool*, which was used to examine the prominent topics in the letters. The *significant building tool* was used to investigate how language and format built up under or decreased the importance of individual subjects. With t*he coordination tool* we examined whether and in what way the letter addressed the involvement of different municipal services, and the hospital staff in the decision-making process. The *relation-building tool* was used to investigate how the letters reflected user involvement and to establish a relationship between the service applicant and the municipality. Following the in-depth analyses of the four tools, we studied how the findings from the tools were inter-related. This contributed to a generation of the main themes of the letters in accordance with the research question [44].

## Results

The analysis led to four main themes presented below: legal letters with formal and comprehensive standardised text; a medically oriented care approach and lack of specification of municipal rehabilitation services; limited coordination of broader municipal services; and user involvement and the role of the applicant – a dual position.

### Legal letters with formal and comprehensive standardised text

By addressing the mandatory elements of the letters in combination with *the subject- and significant tools* we asked what elements the decision contained and how the letters built up or lessened importance of specific topics. When applying these tools to explore the letters in their entirety, it became clear that the letters mostly consisted of a comprehensive and formal standardised text emphasising legal matters. The letters were between two and three pages long, addressed to the applicants and named the municipal decision-making offices as senders. The main body of all the letters consisted of standard text with slight differences between the two individual municipalities. The standard text largely adhered to the mandatory elements in the official guideline, setting out legal regulations and administrative information presented in Table 1.

**Table 1:** Mandatory elements in the individual administrative decision letters

Mandatory elements	Individual administrative decision letters (A- J)									
	**Town municipality**		**City municipality**							
	***A***	***I***	***B***	***C***	***D***	***E***	***F***	***G***	***H***	***J***
**1. Service provision**										
Service to be provided										
In-patient rehabilitation	✓	✓	✓	✓		✓	✓		✓	✓
Practical assistance; ADL training at home					✓					
Long term stay at a housing facility								✓		
Extent of service	**/**	**/**	**/**	✓	**/**	**/**	**/**	**/**	**/**	**/**
Timeframe	Start date open ended	Start date, open ended	✓	✓	✓	✓	✓	✓	✓	✓
**2. Justifications of decision**										
Justification of the decision, including legal references	✓	✓	✓	✓	✓	✓	✓	✓	✓	✓
Facts the decision is based upon	✓	✓	✓	✓	✓	✓	✓	✓	✓	✓
Considerations regarding discretion	✓	✓	✓	✓	✓	✓	✓	✓	✓	✓
**3. Users rights**										
User’s legal rights to access services	✓	✓	✓	✓	✓	✓	✓	✓	✓	✓
User’s right to request deferred implementation of decision	**O**	**O**	✓	✓	✓	✓	✓	✓	✓	✓
User’s right to appeal, deadline, appellate authority and procedure	✓	✓	✓	✓	✓	✓	✓	✓	✓	✓

✓ Element is present in the letter

**/** Element partly addressed

O Element is not present in the letter

Short paragraphs justifying the allocated services included individual-specific information. Up to eighty percent of the content of the letters consisted of standard text. All the letters highlighted the decisions as legally regulated decisions in the headings. Legal aspects were given a dominant position, with legal terms and references throughout the text. The decision concerning service provision was worded in accordance with examples given in the recommendations in the national guideline, as illustrated here:

*“You have been granted a rehabilitation stay in a nursing home, cf. Act on Health and Care Services section 3 − 2 no 6 c. The stay will take place at the xxx rehabilitation and nursing home, the rehabilitation unit from XXX to XXX (dates).”* (Letter B, city municipality)

The letters consisted of general wording pertaining to legal matters such as service costs and the right to appeal the decision:

*“For short-term stays, payment is in accordance with the regulations: ‘User fees for municipal health and care services’ Section 4. Regulation of 16 December 2011, cf. the Municipal Health and Care Services Act § 11 − 2. The rate for short-term stays is NOK 150 per day. If you have had stays lasting for more than 60 days in a calendar year, the municipality may require long-term stay rate in accordance with the regulations: ‘User fees for municipal health and care services’ Section 3.”* (Standard text, town municipality)

*“Appeal: If you do not receive the services, you believe you are entitled to, you may appeal. The appeal is to be submitted to the decision-making office. It will be finally decided by the county governor. You can submit an appeal orally or in writing. In the appeal, you must explain the reason for the appeal and describe which alterations you want. The deadline for appealing is four weeks; see the Patient and User Rights Act Section 7 − 2. The appeal is to be sent to: NN decision-making office.”* (Standard text, city municipality)

Differences between the two municipalities regarding standard texts were found in the opening of the letters. The town municipality started by presenting the decision in a box, followed by the legal justification based on the law, with reference to the municipalities’ responsibility to provide appropriate healthcare services (*Health and Care Services Act Sections 3 − 1 and 3 − 2 no 6 a-d*) followed by the patient and user’s rights (*the Patient and User Rights Act Section 2 − 1 a*). The town municipality used the wording:

“Your application has been received and dealt with in accordance with the Health and Care Services Act Sections 3 − 1 and 3 − 2 no 6 a-d: The municipality must ensure that persons who stay in the municipality are provided with necessary healthcare services. To fulfil this responsibility, the municipality has to provide the following (….)”

The letters further stated that pursuant to the Patient and User Rights Act Section 2 − 1 a:

“Patients and users have a right to necessary health and care services from the municipalities.”

The city municipality started all letters by referring to the Patient and User Rights Act (Section 2 − 1), highlighting that services must be *necessary* and that it must be possible for the municipality to provide help. This was followed by a comment stating that the definition of what is necessary is based on a health and social evaluation and that the provision of services is decided by the office with power of decision in the matter. It was also pointed out that the municipality has limited resources which affects the extend and choice of services offered, but that the services would always cover the applicant’s needs.

### A medically oriented care approach and lack of specification of municipal rehabilitation services

By applying both *the subject- and significant tool* to explore how the letters addressed rehabilitation and the content of services, it became clear that the letters were predominantly medical orientated. All but two letters stated that *rehabilitation* in an in-patient municipal rehabilitation unit was granted. In two deviating decisions, services in form of ‘*Practical assistance with ADL-training at home’* (Letter D, city municipality*) and ‘Long-term stay at a housing facility for young individuals’* (Letter G, city Municipality) were allocated. The latter did not use the term ‘*rehabilitation’* about the allocated services but mentioned the possibility of applying for an in-patient rehabilitation stay upon arrival at the housing facility. None of the letters specified the extent of services by stating their content or frequency of treatment. Differences between municipalities were found regarding the duration of services, in that the town municipality did not state a termination date, while the city municipality set a four-week timeframe in all its letters.

Information about the applicant was provided in a short summary justifying the decision, and all justifications referred to statements made by the hospital rehabilitation departments. All letters granting in-patient rehabilitation included terms such as *progress of recovery* or *potential for further progress* as a rationale. The language was direct and mainly included a medical diagnosis and terms such as the following:

*“Grand cerebral haemorrhage, located in XXX. Resulted in paralysis, speech impairment, swallowing difficulties, visual field loss and neglect. Tried treating with C-PAP treatment, which has had an effect on fatigue, which has been a problem. You have had a PEG inserted, which for a while was the only way for you to get nutrition. Now it is not in particular use, as you eat prepared food BM. There is still a need for further rehabilitation and round-the-clock nursing and care.”* (Letter I, town municipality)

The prior course and progression of recovery was mostly addressed, and, in six letters, the suggested rehabilitation goals focused on body functions:

*“Based on our documents, you are a woman of XX years. Married. Relevant health information: cause of injury, multi-trauma, severe traumatic brain injury. You have received interdisciplinary, specialised rehabilitation for six months. Your functional level is severely reduced, and you need help with all daily activities. You have meanwhile shown progress in some areas and the hospital department evaluates that you are in need of further rehabilitation. The department applies, by agreement with your next of kin, for continuing interdisciplinary rehabilitation in the municipality. It is reported that further purposeful goals will be the following: maintain joint flexibility, avoid contractures, prevent pain due to spasticity, establish yes/no communication, establish non-verbal communication through gestures/mimicry, use right hand in activity.”* (Letter H, city municipality)

Goals referring to cognition were mostly general in their wording:

*“You have had a long rehabilitation stay at the hospital rehabilitation unit. Hospital staff report that you will need full-time care since you still need one-to-one care, in particular due to cognitive difficulties.”* (Letter E, city municipality)

A few letters included specific goals. They addressed specific tasks, such as independence in activities of daily living, e.g. eating meals. Participation or socially oriented care was scarcely mentioned or only in general terms:

*“The hospital department reports that you, especially during the last month, have slowly but surely shown progress. You have an extensive need for help and follow-up in everyday life, but the hospital rehabilitation department considers that you still have a rehabilitation potential, with the goal of i) eating meals, ii) participating more actively in daily activities, iii) clarifying your future housing situation.”* (Letter B, city municipality)

### Limited coordination of broader municipal services

*The coordination tool *was applied to explore whether and in what way the letters addressed the involvement of different municipal services, and the hospital staff in the decision-making process. All the letters stated that the hospital rehabilitation unit took part in the application process and recommended further rehabilitation. Coordination between municipal decision-making offices and specialist health services was evident, as all the letters referred to case investigations, electronic communication, and meetings between the decision-making office and specialist healthcare services. In contrast, there was no explicit mention of how the service providing municipal in-patient rehabilitation participated in the assessment of rehabilitation within the municipal context. Key municipal rehabilitation service providers responsible for separate decisions on service allocation (e.g. speech and language therapy, vision therapy, psychosocial services) were not mentioned in the letters. No information on rights to an individual plan and a coordinator was included.

### User involvement and the role of the applicant: a dual position

By applying *the* relation-building tool the question of how the letters reflected user involvement and established a relationship between the service applicant and the municipality was adressed. The letters provided no direct information about how the applicants or their next of kin participated in the process leading up to the decision on service provision. There were no indications of wishes, concerns or disagreement with the service allocation expressed by the applicant or next of kin that might have been made during the discharge process. A potential collaboration, where the municipalities addressed potential requests from the applicant, were found in a standard statement:

*“The municipality is not bound by the fact that you have applied for a certain type of help, but your wishes will be given weight*.” (Standard text, city municipality)

Only one of the ten letters indicated collaboration between the decision-making office and next of kin. This letter stated that the next of kin wished a specific in-patient institution. The letter did not indicate whether the wish would be taken into account. The letters expressed expectations of user involvement in connection with the individual’s right to appeal and state opinions in upcoming reassessments/changes of needs:

*“New Assessment/Change of need: The need for help will be reassessed. In the event of a change in needs and services, a new administrative decision will be made. You have the right to express your view before a new decision is made, cf. Section 16 of the Public Administration Act*.” (Standard text, town municipality)

The letters contained no information about the names of next of kin or legal guardians, and nor were the letters addressed to them.

The applicant was addressed either by his/her full name and ‘*you’* or in the third person as *‘user’* or *‘patient’*. The letters referring to *‘the patient’* or *‘user’* as well as phrases such as ‘it is the hospital that has applied for you’ conveyed the impression of the applicant being a passive participant. In contrast, phrases like *‘You have the right to’* and *‘you may appeal’* were included in the same letters, denoting the applicant as an active user. Furthermore, expressions such as *‘you must explain’* pointed to responsibilities of the service applicant, placing him/her as an active party with legal possibilities to follow up requirements. The town municipality referred to itself in the third person as *‘the municipality/the decision-making office’*. The city municipality referred to itself as *‘we’*. The terms *‘you’* (the applicant) and *‘*we’ (the municipality) denoted the patients and the municipality as counterparties.

## Discussion

We aimed to examine how municipal decision letters concerning service allocation incorporated the recommendations in the official guideline and reflect a holistic approach to rehabilitation, coordination, and user involvement for individuals with comprehensive needs. The analysis revealed that the letters were of a legal nature, with formal and content-rich standardised text. Individual-specific considerations were included in the justification for the service allocation. They primarily focused on medically oriented care and rarely mentioned activity, participation or psychosocial aspects. Coordination of the municipal services and user involvement were not clearly described.

All the letters contained the required legal references and administrative elements, a finding that is in line with earlier reports [31]. The extent of the standardised reference to legal and administrative regulations seemed noteworthy. This style has been described as a strategy to legitimise professional decisions in different fields of expertise [47]. The legal-administrative focus can be seen as a safeguard to ensure both that the municipalities adhered to the law and that the applicant received services he/she was entitled to. This is important since applicants may not always be capable of asserting their own rights. The individual justifications in the letters illustrated that this was the case in our study, as they indicate that many of the patients needed help to meet their fundamental needs (e.g. nutrition, communication, cognition). Nevertheless, as the letters mainly consisted of legal and administrative text and references combined with medical jargon, this may affect the readability for the user and their next of kin.

A key finding with regard to service allocation was that the service was not specified beyond the term *rehabilitation.* This contrasts with the guideline, which state that decision letters should as far as possible be designed in such a way that the applicant understands what services are to be provided and their scope. This can be crucial for several reasons. The allocation of *rehabilitation* may appear transparent and fair, since applicants differ and can receive the services they need based on individual evaluations by those involved in their treatment. However, since it is far from clear what the term rehabilitation encompasses, even for specialists [12], this may be a pitfall.

While the justification and goals stated in individual letters can serve as indicators of/references to specific needs, it leaves open questions such as who (profession) will provide the rehabilitation, when and how often. For individuals and their next of kin, this may be a crucial issue as they link this information to the chance of recovery. This lack of specification left them in a state of uncertainty and may increase the burden of care. They not only have to adapt quickly to a life-changing situation, but also to a healthcare system where each new stage of the system requires a lot of information and effort to understand [15, 16]. The lack of specification concerns both the applicants’ right to appeal the decision and the notification of a reassessment of needs. How can they appeal a decision when they do not know what its content is (the allocated rehabilitation services*)* either upon discharge or after reassessment? It has been suggested that an undetermined conceptualisation of rehabilitation changes the focus from the content to the management of the services and that this will ultimately lead to a downscaling of services [5]. These findings call for more research highlighting how rehabilitation is conceptualised by policymakers, the individuals concerned and their next of kin.

The issue of coordination of different municipal service-providing units is related to the lack of specification of rehabilitation. Previous research has demonstrated that several decisions concerning service allocation in comprehensive rehabilitation pathways are made by different service providers (e.g. offices that make decisions on in-patient rehabilitation, speech and language therapy or psychological services) [37]. The guideline published by the Norwegian Directorate of Health in 2016 [28] suggests that listing all relevant municipal services in one and the same decision letter can provide an overview when several services are involved. The analysed decision letters did not mention other services, such as speech and language therapy or social services. Hence, the applicant was not given an overview, and potential coordination efforts remained unclear. Further, none of the letters included information about the right to a coordinator or an individual plan. Hence, the letters appear to be a tool for informing about an in-patient rehabilitation stay, rather than a tool for informing applicants about coordination and coherent municipal services. This seems to be in contrast to the intentions and goals of the healthcare reform [25]. Norwegian municipalities are free to design their services and to differ as regards the services they provide and in terms of the healthcare staff’s specialisations and experience of treating individuals with complex needs [49]. Therefore, the use of the generic term ‘rehabilitation’ gave little indication of what services and professions are allocated and coordinated. Considerations of efficacy and equality were not evident in the decision letters.

Another key finding was that the letters prioritised medically oriented care (disease and symptoms), whereas psychosocial aspects, including activity and participation, were mentioned in general terms or missing. Since the participants were moderately or even severely injured and needed help with basic needs, this may not be surprising [11, 15, 50]. Reading individual information in the justification and goals set out in the letters, it seems that they were to a great extent derived from specialist health care. It is unknown to what extent psychosocial issues were addressed before discharge from hospital. However, it remains an issue that the term psychosocial is used in policy documents without more detailed specification [32]. As several authors have stressed, individuals with complex needs are a vulnerable group with social needs over and above managing treatment and medicines. Social participation, activities and relationships are important to address across sectors [14, 32, 50]. In addition to psychosocial needs, user involvement is seen as imperative in political documents and in the healthcare and rehabilitation service [5, 23, 25]. Hence, one striking feature of the decision letters is the absence of any clear indication of how the applicant and next of kin were involved in the decision-making process. Previous research has also identified that the individuals in question lack a voice in both medical and legal written documents [46, 51], and several authors have stressed that individuals might not be able to be active or have to be helped to be active and make decisions for themselves [5, 14, 23]. These and our findings suggest that user involvement does not seem to have gained the position in the municipalities as the national policy intended.

In the present study, the letters did not seem to establish a joint, collaborating ‘we’ between the municipality and the applicant that could develop and carry out a common rehabilitation process. Rather, the letters positioned the applicant and the municipality as counterparts. The structure and wording of the letters gave the impression of an authoritative approach to the applicant, while the applicant was placed in a dual position. On the one hand, the applicant was addressed as active and knowledgeable with the capability to appeal, pay for and manage transport and the work environment of service providers [5, 23, 24]. On the other hand, he/she was a passive recipient who had comprehensive difficulties managing everyday activities, cognition, and communication. Solholt and Frederiksen [23] argued that, although many patients may benefit from having an active role, this approach requires an active, knowledgeable patient who has insight into the disease and supportive relatives, and that there is hardly room for a passive patient. This raises the question of whether the ideal of an active patient may disguise the user’s vulnerable position and, despite the intentions, potentially leave severely disabled and chronically ill patients deprived and vulnerable [5, 23]. It is necessary, to ensure that their rights are secured, to focus further on how to address user involvement in instances where the users may have difficulties playing an active role.

The analysis revealed a clear focus on legal and administrative issues in official letters that were in accordance with the guideline. The findings demonstrate that the content of the rehabilitation was not further specified. The letters show that the allocation of services was primarily focused on medically oriented care, with a shifting perception of the user as both active or passive, and limited coordination of services. In the discourse on rehabilitation, there has been a development towards a broader understanding of rehabilitation, including a holistic view of people’s needs (beyond a medical focus), the coordination of services, and user involvement. However, the findings show that these elements are lacking in the letters describing rehabilitation for these patients, while transitioning from hospitals to municipalities. Not describing these central issues may increase the possibility that service providers will not meet individual needs, which, in turn, can result in a downscaling of services or reduction in quality. Those allocating services may be aware of these discrepancies, but be in a challenging position where they have to strike a balance between the demands of the national guideline and municipal prioritisations and resources, as well as manoeuvring around organisational barriers within a tight framework.

### Strengths and limitations

Discourse analysis is an interpretive process, and we recognise that other questions could have provided other perspectives [43]. We argue that the strength of such an approach is that the selection of questions and analytical exploration of the topics are transparent. We further acknowledge that the number of decision letters is small and that they concern a specific user group. However, we argue that the strategic selection makes them well-suited for conducting thorough discourse analyses. The letters analysed are from a Norwegian context. Yet, we argue that this analysis contributes to the broader international discussion on the implementation of national policy in municipal practice.

When analysing written text, it is important to recognise that what is written may not fully reflect the processes relating to service allocation. As such, analyses of decision letters only give partial insight into how a municipality addresses rehabilitation, user involvement and coordination in the allocation of services. We argue, however, that these letters play a fundamental role in the service allocation process and are, as such, worth analysing. Further research is needed to gain more insight into how policy and the municipalities address rehabilitation, user involvement, service involvement and coordination in the allocation of services in rehabilitation pathways.

## Conclusions

Decision letters are intended to implement national policy for the allocation of healthcare services. They fulfil legal and administrative recommendations concerning service allocation, and legal and administrative issues have a dominant influence on the wording of the decision letters. Despite the intentions, however, the applicants are left in a dual position where they are responsible for exercising their legal rights and fulfilling obligations that are hard to grasp with little information about the content of services. The decision letters did not fulfil their potential to serve as a means of conveying rehabilitation issues, such as a holistic approach to health, coordination, or the involvement of users in decision processes, which must be acknowledged as essential. These elements are important to incorporate throughout the allocation process if policies are to be implemented as intended.

## Data Availability

The dataset for this study is not publicly available due to the sensitivity of the material. It may be made available from the correspondent author on reasonable request.
